# Repetitive Elements, Sequence Turnover and Cyto-Nuclear Gene Transfer in Gymnosperm Mitogenomes

**DOI:** 10.3389/fgene.2022.867736

**Published:** 2022-05-25

**Authors:** Hui Liu, Wei Zhao, Ren-Gang Zhang, Jian-Feng Mao, Xiao-Ru Wang

**Affiliations:** ^1^ National Engineering Laboratory for Tree Breeding, Key Laboratory of Genetics and Breeding in Forest Trees and Ornamental Plants, Ministry of Education, The Tree and Ornamental Plant Breeding and Biotechnology Laboratory of National Forestry and Grassland Administration, College of Biological Sciences and Technology, Beijing Forestry University, Beijing, China; ^2^ Department of Ecology and Environmental Science, Umeå Plant Science Centre, Umeå University, Umeå, Sweden; ^3^ Department of Bioinformatics, Ori (Shandong) Gene Science and Technology Co., Ltd., Weifang, China

**Keywords:** *Platycladus orientalis*, gene transfer, gymnosperm mitogenome, rearrangement, repetitive sequences, substitution rate, sequence turnover, RNA editing

## Abstract

Among the three genomes in plant cells, the mitochondrial genome (mitogenome) is the least studied due to complex recombination and intergenomic transfer. In gymnosperms only ∼20 mitogenomes have been released thus far, which hinders a systematic investigation into the tempo and mode of mitochondrial DNA evolution in seed plants. Here, we report the complete mitogenome sequence of *Platycladus orientalis* (Cupressaceae). This mitogenome is assembled as two circular-mapping chromosomes with a size of ∼2.6 Mb and which contains 32 protein-coding genes, three rRNA and seven tRNA genes, and 1,068 RNA editing sites. Repetitive sequences, including dispersed repeats, transposable elements (TEs), and tandem repeats, made up 23% of the genome. Comparative analyses with 17 other mitogenomes representing the five gymnosperm lineages revealed a 30-fold difference in genome size, 80-fold in repetitive content, and 230-fold in substitution rate. We found dispersed repeats are highly associated with mitogenome expansion (*r* = 0.99), and most of them were accumulated during recent duplication events. Syntenic blocks and shared sequences between mitogenomes decay rapidly with divergence time (*r* = 0.53), with the exceptions of Ginkgo and Cycads which retained conserved genome structure over long evolutionary time. Our phylogenetic analysis supports a sister group relationship of Cupressophytes and Gnetophytes; both groups are unique in that they lost 8–12 protein-coding genes, of which 4–7 intact genes are likely transferred to nucleus. These two clades also show accelerated and highly variable substitution rates relative to other gymnosperms. Our study highlights the dynamic and enigmatic evolution of gymnosperm mitogenomes.

## 1 Introduction

In contrast to the ∼8,600 chloroplast (https://www.ncbi.nlm.nih.gov/) and ∼800 nuclear reference genomes assembled (Marks et al., 2021) for land plant taxa thus far, only ∼350 mitogenomes have been released (https://www.ncbi.nlm.nih.gov/; by November 2021). These published plant mitogenomes revealed extensive diversity in genome size, genome rearrangement, nucleotide substitution rate, and gene contents (Palmer and Herbon, 1988; Chaw et al., 2008; Smith and Keeling, 2015; Mower, 2020; Wu et al., 2020). In angiosperms, mitogenomes span a 170-fold range in size, from ∼66 Kb in *Viscum scurruloideum* to ∼11.3 Mb in *Silene conica* (Sloan et al., 2012; Skippington et al., 2015). The mechanisms contributing to the size variation are hypothesized to include horizontal transfer, intergenomic transfer, and repetitive sequence proliferation (Goremykin et al., 2012; Rice et al., 2013; Guo et al., 2017). For example, the mitogenome of the basal angiosperm *Amborella trichopoda* acquired ∼20% of its sequences through horizontal transfer from green algae, mosses, and other plants (Rice et al., 2013), while apple and maize acquired 20–78% of mitochondrial DNA (mtDNA) from their nuclei (Goremykin et al., 2012). The repeat content in 82 angiosperm mitogenomes ranged from 1.3% to 48.9% (Dong et al., 2018). A phylogenetic pattern in these traits is, however, not yet discernable.

Plant mitogenomes are often characterized by a strong dichotomy between rates of sequence and structural evolution (Palmer and Herbon, 1988). Analyses of the available mitogenomes show that while most plant lineages have low substitution rates, it is misleading to categorize all plants in this way; synonymous substitution rates are exceptionally variable, with four orders of magnitude across seed plants (Mower et al., 2007). The reason for such fluid substitution rates remains unclear.

Gymnosperms contain ∼1,000 species representing five of the six main lineages of seed plants, including Cupressophytes, Pinaceae, Gnetophytes, Ginkgo, and Cycads (Wang and Ran, 2014). Up to now, only ∼20 gymnosperm mitogenomes have been released, which are too few to allow a proper understanding of the properties and diversities of this genome. Despite the limited data, interesting and sometimes unexpected patterns of mtDNA variation have emerged. Similar to angiosperms, gymnosperm mitogenomes also show a wide range in size, with *Ginkgo biloba* (∼347 Kb) and *Larix sibirica* (∼11.7 Mb) being the extremes (Guo et al., 2016; Putintseva et al., 2020). The driving factors for this size heterogeneity are, however, debated because estimations of repetitive contents and nucleus-derived sequences vary among studies (Guo et al., 2016; Kan et al., 2020; Sullivan et al., 2020).

Fast structural rearrangement of gymnosperm mitogenomes is reported to result in a substantial loss of synteny and shared sequences between closely related species with divergence times as small as 15 million years ago (MYA) (Guo et al., 2016; Cole et al., 2018; Sullivan et al., 2020). On the other hand, conserved structures and high degrees of synteny are found between *Cycas* and *Ginkgo* that diversified >300 MYA (Guo et al., 2016). Parallel to this unpredictable structural diversity are the highly variable substitution rates among the taxa (Guo et al., 2016; Sullivan et al., 2020; Kan et al., 2021). In addition, the number of protein-coding and other genes vary between gymnosperm lineages (Guo et al., 2020; Kan et al., 2021). These observations show a largely lineage-specific mode of mitogenome evolution in gymnosperms, but the mechanisms and phylogenetic patterns for this diversity are unclear due to sparse sampling and limited analyses.

In this study, we report a complete mitogenome assembly of *Platycladus orientalis*, Cupressaceae (Cupressophytes). Cupressaceae is the largest conifer family with 25 genera; *Platycladus* is a monotypic genus in this family. This new genome thus fills a gap in the still sparse coverage for this clade. We then sampled 17 gymnosperm and two angiosperm mitogenomes, representing all six major lineages of seed plants, to characterize gene content, transposable elements (TEs), dispersed repeats, tandem repeats, sequence turnover, structural rearrangement and substitution rate variation. We provide quantitative estimates of the role of repetitive elements in genome expansion, the mode of repeats accumulation, and the rate of synteny decay among taxa. Our results reinforce some early observations but also shed new light on the dynamics of mitogenome evolution.

## 2 Materials and Methods

### 2.1 Mitochondrial Genome Assembly and Annotation

We collected fresh young leaves of *P. orientalis* from an elite tree in a seed orchard in Jiaxian, Henan Province, China. DNA used for Illumina sequencing was isolated using a cetyl trimethyl ammonium bromide (CTAB)-based method (Doyle and Doyle, 1987). For the Oxford Nanopore Technologies (ONT) sequencing, we prepared DNA using a DNeasy Plant Mini Kit (QIAGEN). A short-insert 2 × 150 bp pair-end library was constructed following the manufacturer’s PCR-free protocol (Illumina) and sequenced on an Illumina NovaSeq 6000 platform. Library for ONT sequencing was prepared following the Nanopore 1D Genomic DNA by ligation (SQK-LSK109)–PromethION protocol, and sequenced on a PromethION platform. The short reads from Illumina were filtered using fastp v0.21.0 (Chen et al., 2018) with parameters of “-q 20 -l 36 --cut_right --n_base_limit 0 -c.” The long reads from Nanopore were corrected using NextDenovo v1.1.0 (https://github.com/Nextomics/NextDenovo). We generated ∼110 Gb Illumina clean short reads and ∼808 Gb (40.3 M reads with a N50 of 25 Kb) corrected ONT long reads.

We applied a long and short reads hybrid strategy to assemble the *P. orientalis* mitogenome. First, the clean short reads were *de novo* assembled using GetOrganelle v1.6.4 (Jin et al., 2020) with parameters of “-R 50 -k 67,87,107,127 -F embplant_mt --reduce-reads-for-coverage inf -w 127 -t 12 --max-reads 7.5E8”. The resulting unitig graph was manually edited using Bandage v0.8.1 (Wick et al., 2015) to remove chloroplast- and nuclear-derived unitig nodes. The corrected long reads were then mapped to the graph using the minimap2 v2.24-r1122 (Li, 2018). Finally, repeats on the graph were resolved by aligning with the mapped long reads.

The protein-coding genes, non-coding rRNA, and tRNA genes were annotated using the Organelle Genome Annotation Pipeline (OGAP, https://github.com/zhangrengang/OGAP) with parameters of “-taxon Acrogymnospermae Bryophyta Liliopsida commelinids fabids malvids -mt -trn_struct”. More specifically, protein-coding genes were identified with Exonerate v2.2.0 (Slater and Birney, 2005) and AUGUSTUS v3.3.3 (Stanke et al., 2008), tRNA genes with tRNAscan-SE v2.0.5 (Lowe and Eddy, 1997), and rRNA genes with BLAT v36x2 (Kent, 2002) within the OGAP.

RNA editing sites in protein-coding genes of each species were predicted using the PREP-Mt (Mower, 2005) with a cutoff of 0.2. To validate the accuracy of the predictions, we searched for RNAseq data of *P. orientalis* (Supplementary Table S1) and mapped them to the protein-coding genes with bwa mem v0.7.17-r1188 (Li, 2013) to determine the edited sites using REDItoolDenovo v1.3 (Lo Giudice et al., 2020) with minimum coverage of 10 and mapping quality score of 30.

### 2.2 Repetitive Sequences

To estimate the repetitive sequences in mitogenomes, we annotated tandem repeats, TEs, and dispersed repeats in *P. orientalis* and 19 other taxa representing the six major lineages of seed plants included in this study (Table 1). Tandem repeats were identified using Tandem Repeats Finder v4.10.0 with the parameters of “2 7 7 80 10 50 800 -d” (Benson, 1999). For TEs, we first used the *de novo* TE family identification and modeling package RepeatModeler v1.0.10 (Smit and Hubley, 2008) to train a TE database from each mitogenome. The RepeatModeler derived library was then combined with the database “RepeatMasker.lib” of RepeatMasker v4.07 (Smit et al., 2013) and the repeat library of *Picea abies* v1.0 assembly (Nystedt et al., 2013) to generate a joint repeats library. Finally, TEs in each genome were identified by RepeatMasker using this reference database.

**TABLE 1:** Summary of the 20 plant mitogenomes.

Lineage	Species	Genome size (Kb)	No. scaffolds	GC (%)	Accession number	References
Cupressophytes	*Platycladus orientalis*	2,624	2	50.60	OL703044-45	This study
Cupressophytes	*Cupressus sempervirens*	2,743	238	50.64	MN965161-90	Guo et al. (2020)
Cupressophytes	*Hesperocyparis glabra*	1,967	129	50.02	MN965210-41	Guo et al. (2020)
Cupressophytes	*Taxus cuspidata*	469	1	50.39	MN593023	Kan et al. (2020)
Cupressophytes	*Podocarpus macrophyllus*	2,021	85	47.44	MN965338-58	Guo et al. (2020)
Cupressophytes	*Araucaria heterophylla*	1,384	52	46.80	MN965108-21	Guo et al. (2020)
Gnetophytes	*Gnetum gnemon*	640	44	47.88	MN965191-209	Guo et al. (2020)
Gnetophytes	*Welwitschia mirabilis*	979	1	53.02	NC_029130	Guo et al. (2016)
Pinaceae	*Picea abies*	4,899	4	44.67	MN642623-626	Sullivan et al. (2020)
Pinaceae	*Picea glauca*	5,995	36	44.67	LKAM00000000	Jackman et al. (2015)
Pinaceae	*Pinus lambertiana*	4,021	262	44.91	NA	Wegrzyn et al. (2008)
Pinaceae	*Pinus taeda*	1,191	1	46.67	NC_039746	NA
Pinaceae	*Larix sibirica*	11,663	9	41.90	MT797187-95	Putintseva et al. (2020)
Pinaceae	*Abies sibirica*	1,485	237	45.73	MN965088-107	Guo et al. (2020)
Ginkgo	*Ginkgo biloba*	347	1	50.36	NC_027976	Guo et al. (2016)
Cycads	*Ceratozamia hildae*	412	85	47.54	MN965122-60	Guo et al. (2020)
Cycads	*Zamia integrifolia*	469	96	47.59	MN965050-87	Guo et al. (2020)
Cycads	*Cycas taitungensis*	415	1	46.92	NC_010303	Chaw et al. (2008)
Angiosperm	*Amborella trichopoda*	3,866	5	45.92	KF754799-803	Rice et al. (2013)
Angiosperm	*Liriodendron tulipifera*	554	1	47.70	KC821969	Richardson et al. (2013)

Dispersed repeats were identified with a BLASTN search using a word size of seven and an e-value of 1*E*-6 as in a previous study (Guo et al., 2016). Overlapping regions of dispersed repeats were counted only once when calculating their total size. The dispersed repeats were grouped into small-, medium-, and large-size classes with sequence lengths of <100 bp, 100–1,000 bp, and ≥1,000 bp, respectively. Divergence among repeats was estimated using Kimura two-parameter distance (K2P) (Kimura, 1980) for pairwise repeat sequences.

### 2.3 Identify Mitochondrial Genes From Transcriptomes

Based on gene annotation results, we found 8–12 genes were absent in mitogenomes of Cupressophytes and Gnetophytes (see Section 3.3). To investigate the possibility of their transfer to nuclei, we first tried to recover these genes from transcriptomes. We searched for RNAseq data of Cupressophytes and Gnetophytes in NCBI (https://www.ncbi.nlm.nih.gov/), and *de novo* assembled transcriptomes for *Cupressus sempervirens*, *Hesperocyparis glabra*, *Taxus cuspidata*, *Podocarpus macrophyllus*, *Gnetum gnemon*, and *Welwitschia mirabilis* (Supplementary Table S2)*.* The Illumina reads were filtered using fastp v0.21.0 (Chen et al., 2018) with the parameters of “-q 20 -l 36 --cut_right --n_base_limit 0 -c,” generating 6.3–14.7 Gb clean reads per species. We then *de novo* assembled the transcriptomes using Trinity v2.8.5 with default parameters (Grabherr et al., 2011). The transcriptome of *P. orientalis* is from our early study (Hu et al., 2016). For *Araucaria heterophylla*, we only found 212 ESTs/cDNAs in TreeGenes database (Wegrzyn et al., 2008) (Supplementary Table S2). We searched for intact mitochondrial genes in these transcriptomes and ESTs/cDNAs using OGAP with the parameters of “-taxon Acrogymnospermae Bryophyta Liliopsida commelinids fabids malvids -mt -trn_struct -trans”. Potential chloroplast genes were filtered out after BLAST against NCBI’s NT database (*E*-value < 1*E*-40, sequence coverage >99%, and sequence identity >99%).

Second, to confirm that the transcriptome-recovered mitochondrial genes (mitogenes) were in nuclear genomes, we used TBLASTN to search for matches of these genes in reference genomes of Cupressophytes and Gnetophytes, including *Sequoiadendron giganteum* (Scott et al., 2020), *Sequoia sempervirens* (Neale et al., 2021), *Taxus chinensis* (Xiong et al., 2021), *Taxus wallichiana* (Cheng et al., 2021), *Gnetum montanum* (Wan et al., 2018), and *Welwitschia mirabilis* (Wan et al., 2021) (Supplementary Table S3).

Third, we used mapping depth of reads of resequencing data to verify the genome origins of the transcriptome-recovered mitogenes. In a plant cell, the abundance of nuclear and organelle genomes can differ by thousands fold with the copy numbers of plastid the highest, mitochondrial intermediate and nuclear the lowest (Petit and Vendramin, 2007). Based on this expectation, we investigated the depth of read coverage of each recovered genes. The mitochondrial genes retrieved from mitogenome and transcriptomes were used as reference. We also include the low-copy nuclear gene *LEAFY* and chloroplast gene *matK* as spike-in reference. We downloaded whole-genome resequencing data of *Cupressus sempervirens*, *Hesperocyparis glabra*, *Taxus cuspidata*, *Podocarpus macrophyllus*, *Gnetum gnemon*, and *Welwitschia mirabilis* from Sequence Read Archive (SRA) of NCBI (https://www.ncbi.nlm.nih.gov/sra) (Supplementary Table S4), the resequencing data of *P. orientalis* is from this study. The Illumina reads were filtered using fastp v0.21.0 (Chen et al., 2018) with the same parameters as in Section 2.1, generating 7.2–31.4 Gb clean reads per species. The clean reads were mapped to the reference genes using bwa mem v0.7.17-r1188 with default parameters. The resulting sequence alignment maps (SAMs) were sorted with SAMtools v1.14 (https://github.com/samtools/samtools), reads with alignment score (AS) <25 were removed by using ngsutilsj (https://github.com/compgen-io/ngsutilsj). We used SAMtools to compute the depth of the aligned reads at each position of the gene.

### 2.4 Genome Alignments and Shared DNA Blocks

To identify synteny blocks among the mitogenomes of the major gymnosperm lineages, we performed pairwise genome alignment using Mauve v2.4.0 with default parameters (Darling et al., 2010). To calculate the degree of shared DNA between species, each pair of mitogenomes were aligned using BLASTN with a word size of seven and an *E*-value of 1*E*-6.

### 2.5 Phylogenetic Analysis and Estimation of Nucleotide Substitution Rates

We used 28 protein-coding genes shared among all 20 mitogenomes included in this study to reconstruct a phylogenetic tree. Edited protein sequences were individually aligned with MAFFT v7.453 with default parameters and converted into the corresponding edited codon sequence alignments. Poorly aligned regions were trimmed using BMGE v1.1 (Criscuolo and Gribaldo, 2010) with parameters of “-t CODON -g 0.5.” The trimmed alignments of each gene were concatenated into a single alignment of 26,844 bp. This alignment was used to build a maximum-likelihood (ML) phylogenetic tree using IQ-TREE v1.6.12 (Nguyen et al., 2015), using the best-fit model TVM + F + R3 selected by ModelFinder (Kalyaanamoorthy et al., 2017) and with 1,000 replications of ultrafast bootstrap and Shimodaira-Hasegawa-like approximate likelihood-ratio (SH-aLRT) test. The branch lengths in units of synonymous (d*S*) and nonsynonymous (d*N*) substitution rates were estimated under the free-ratio branch model using codeml in PAML v4.9j package (Yang, 2007).

To estimate the divergence time between lineages, we run the MCMCTree in PAML v4.9j (Yang, 2007) for 2,000,000 iterations and 100,000 iterations for a burn-in. To calibrate the tree, we used the following time points of diversification from the TimeTree web (http://www.timetree.org/): 289–337 MYA between gymnosperms and angiosperms, 282–324 MYA between Cycads and other gymnosperm clades, 159–246 MYA between *Cycas taitungensis* and the other Cycads species, 134–197 MYA between *Abies sibirica* and other species within Pinaceae, 7.6 to 30.7 MYA between *Picea abies* and *Picea glauca*, 59–87 MYA between *Pinus taeda* and *Pinus lambertiana*, 99–123 MYA between *Gnetum gnemon* and *Welwitschia mirabilis*, and 213–272 MYA between the clades consisting of *Podocarpus macrophyllus* and *Araucaria heterophylla* and the other species of Cupressophytes.

## 3 Results

### 3.1 The Mitogenome of *P. orientalis*

**FIGURE 1:**
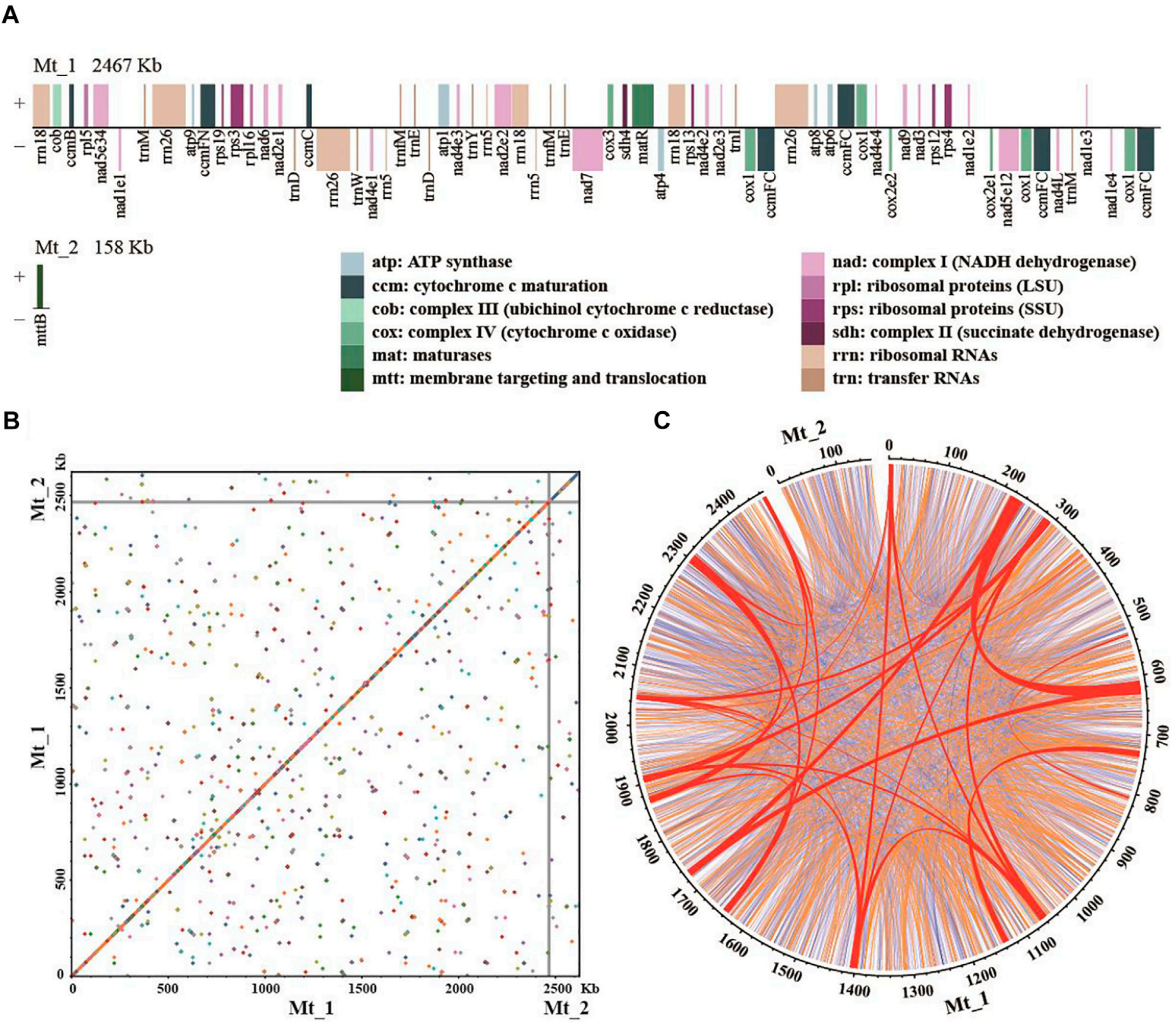
The mitogenome assembly of *Platycladus orientalis.* **(A)** The order, orientation, and size of genes on the two chromosomes of *P. orientalis* mitogenome. The *nad1*, *nad2*, *nad4*, *nad5*, and *cox2* are *trans*-splicing genes. Each box is proportional to the size of the gene including introns; genes with <200 nucleotides are shown as 200 nucleotides. Intergenic regions are not to scale. **(B)** The dot plot shows self-alignment. Dots represent the homology sequences with length >100 bp. **(C)** Distribution of dispersed repeats. Large-size repeats ≥1,000 bp in length are indicated in red, medium-size repeats in the range of 100–1,000 bp are in orange, and small-size repeats <100 bp are in blue. The numbers on the ring anchor genome coordinates in kilobases.

We assembled the mitogenome of *P. orientalis* into two circular-mapping chromosomes representing a master ring and a sub-genomic ring with a total size of ∼2.6 Mb (Figure 1A, Table 2). This genome lacks large structural variants but is rich in dispersed repeats (Figures 1B,C). The genome size is similar to *C. sempervirens* (2.7 Mb) and *H. glabra* (2.0 Mb) in the family of Cupressaceae, but more than 5-fold larger than *T. cuspidata* and ∼2–4 fold smaller than *Picea* and *Larix* in Pinaceae (Table 1). Across the Cupressophytes and Pinaceae, the mitogenomes vary 6–10 fold in size, in contrast to the relatively stable and small genomes in Cycads and Gnetophytes.

**TABLE 2:** Characteristics of the *Platycladus orientalis* mitogenome.

Feature	Values
Genome size	2,624 Kb
GC content	50.6%
Protein-coding genes	32 (41 Kb)
*cis*-spliced introns	5
*trans-*spliced introns	10
Predicted RNA editing sites	1,007
Observed RNA editing sites	1,068
tRNAs	7
rRNAs	3
Chloroplast genes	9 (6 Kb)
Repetitive sequences	608 Kb (23.2%)
Tandem repeats	123 Kb (4.7%)
Transposable elements (TEs)	157 Kb (6.0%)
Dispersed repeats	541 Kb (20.6%)
Undefined sequences	1,983 Kb (75.6%)

**FIGURE 2:**
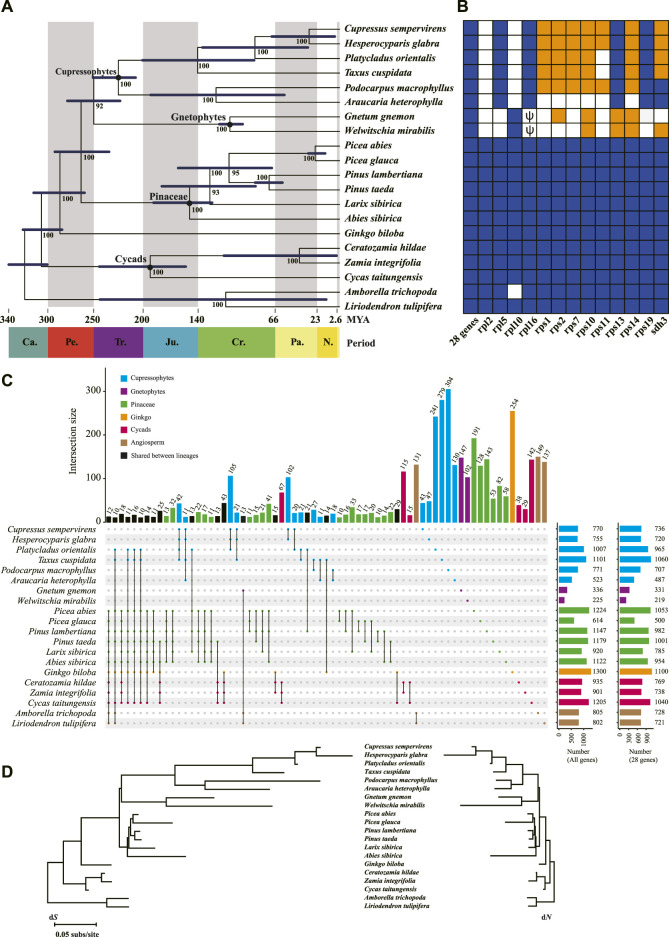
Phylogeny and gene content of the 20 mitogenomes included in this study. **(A)** The phylogenetic tree among major gymnosperm lineages based on 28 shared genes. Numbers next to nodes indicate bootstrap values from 1,000 replicates, and blue bars correspond to the 95% highest posterior density (HPD) of divergence time. Period is shown at the bottom of the tree. Ca., Carboniferous; Pe. Permian; Tr. Triassic; Ju. Jurassic; Cr. Cretaceous; Pa. Paleogene; N. Neogene. **(B)** Gene contents in the 20 mitogenomes. The 28 shared genes are *atp*[1, 4, 6, 8, 9], *ccm*[*B*, *C*, *Fc*, *Fn*], *cob*, *cox*[1–3], *matR*, *mttB*, *nad*[1, 2, 3, 4, 4L, 5, 6, 7, 9], *rps*[3, 4, 12], and *sdh*4. Intact genes are indicated by dark blue blocks and missing genes in white. The lost genes that are confirmed as transferred to nucleus are highlighted in orange. Pseudogenes are indicated as ψ. **(C)** RNA editing sites in the 20 mitogenomes. The two plots in the far right show the number of editing sites for all genes and the 28 shared genes, respectively. **(D)** Nucleotide substitution rates in the 20 mitogenomes. Branch lengths are proportional to rates of synonymous (d*S*) and nonsynonymous (d*N*) substitutions inferred by the maximum-likelihood method implemented in PAML.

The mitogenome of *P. orientalis* contains 32 distinct protein-coding genes (Figure 1A, Table 2), seven tRNA genes (*Asp-trnD-GUC*, *Glu-trnE-UUC*, *Ile-trnI-CAU*, *Met-trnM-CAU*, *Met-trnfM-CAU*, *Trp-trnW-CCA*, and *Tyr-trnY-GUA*), and three rRNA genes. Among them, *ccmFC* and *cox1* have four copies, *rrn5*, *rrn18*, and *rrn26* have three copies, *nad1*, *trnD-GUC*, *trnE-UUC*, *trnM-CAU*, and *trnfM-CAU* have two copies. We found five *cis*-spliced introns and 10 *trans*-spliced introns in the protein-coding genes (Supplementary Table S5). Both the number of protein-coding genes (Figure 2B) and the number of *cis*- and *trans*-spliced introns identified in *P. orientalis* are similar to that of Cupressophytes taxa reported previously (Guo et al., 2020). We also detected seven complete chloroplast genes (although they may not be functional), *atpA*, *ndhA*, *ndhB*, *ndhC*, *ndhD*, *ndhE*, and *ndhH,* and two chloroplast pseudogenes, *petB* and *petD,* in the mitogenome of *P. orientalis* (Table 2).

### 3.2 RNA Editing, Mitochondrial Genes-Based Phylogeny, and Variable Substitution Rates in Cupressophytes and Gnetophytes

RNA editing remodels variability in higher plant mitogenomes, and could bias phylogenetic inferences if not accounted for (Szmidt et al., 2001; Mower, 2005). We predicted the RNA editing sites in protein coding genes of all 20 species and found that Ginkgo (1,300 sites) and Gnetophytes (225–336 sites) had the highest and lowest C-to-U editing sites across all protein-coding regions, respectively, and the other species spanned a range of 520–1,220 sites (Figure 2C). This variation in editing frequency reflects a lineage-specific pattern of editing, for example 25% (254) and 44–45% (102–147) of the editing sites in Ginkgo and Gnetophytes were unique (Figure 2C). In contrast, *C. sempervirens* and *H. glabra* of Cupressophytes each had only 6% (43–47) unique sites (Figure 2C).

To assess the accuracy of the predicted editing sites, we identified the edited sites from RNAseq data in *P. orientalis*. A total of 1,068 C-to-U edit sites were detected in 32 genes, of which 745 sites were among the 1,007 predicted sites (Table 2, Supplementary Table S6). This comparison suggests that the prediction method provides a reasonably reliable estimate of RNA editing (74% in *P. orientalis*), consistent with the results from a previous study (Guo et al., 2016).

We inferred the phylogeny of the major gymnosperm lineages using 28 conserved protein-coding genes common to all 20 species included in this study. The concatenated sequence alignment consisted 26,844 sites. On this phylogenetic tree, Cupressophytes and Gnetophytes are sister clades with a strong bootstrap support of 92% (Figure 2A). Within Cupressophytes, *P. orientalis* is sister to the group consisting of *C. sempervirens* and *H. glabra* (Figure 2A), and diverged from this group 87 MYA in the Paleogene. The divergence of the Cupressaceae family is dated to 145 MYA (Figure 2A), which is similar to the 159 MYA estimate from nuclear data (De La Torre et al., 2017).

To evaluate the substitution rates in mitochondrial genes, we estimated synonymous (d*S*) and nonsynonymous (d*N*) substitution rates along the branches of the inferred phylogeny. We found that d*S* and d*N* varied up to 46- and 234-fold among species, respectively. This wide range is mainly caused by Cupressophytes and Gnetophytes with their substantially longer branches and larger variation in d*S* and d*N* among species (Figure 2D; Table 3) compared to the other clades, all of which have low substitution rates. The overall d*S* of Cupressophytes and Gnetophytes is more than 5-fold higher than Pinaceae. The same overall pattern is seen with d*N*, although it is about 20–70% of the d*S* values, reflecting selective constraints on nonsynonymous mutations (Figure 2D; Table 3). Within Cupressophytes, d*S* varied by 46-fold and d*N* varied by 25-fold among lineages, with *P. orientalis* at the low end for both values (d*S =* 0.0030, d*N* = 0.0015; Table 3).

**TABLE 3:** Relative (d*S*, d*N*) and absolute (R*S*, R*N*) synonymous and nonsynonymous substitution rates. MYA, million years ago.

Species	Time (MYA)	d*S*	R*S* (site/year)	d*N*	R*N* (site/year)
*Platycladus orientalis*	87	0.002997	3.44483E-11	0.001498	1.72184E-11
*Cupressus sempervirens*	31	0.004554	1.46903E-10	0.003375	1.08871E-10
*Hesperocyparis glabra*	31	0.042915	1.38435E-09	0.037602	1.21297E-09
Cupressaceae	145	0.050927	3.51221E-10	0.016829	1.16062E-10
*Taxus cuspidata*	145	0.037293	2.57193E-10	0.014685	1.01276E-10
*Podocarpus macrophyllus*	127	0.137564	1.08318E-09	0.017518	1.37937E-10
*Araucaria heterophylla*	127	0.077235	6.08150E-10	0.019114	1.50504E-10
**Cupressophytes**	**252**	**0.064140**	**2.54524E-10**	**0.018398**	**7.30079E-11**
*Gnetum gnemon*	112	0.057119	5.09991E-10	0.027154	2.42446E-10
*Welwitschia mirabilis*	112	0.126746	1.13166E-09	0.073152	6.53143E-10
**Gnetophytes**	**252**	**0.053585**	**2.12639E-10**	**0.019649**	**7.79722E-11**
*Picea abies*	25	0.005778	2.31120E-10	0.005871	2.34840E-10
*Picea glauca*	25	0.046808	1.87232E-09	0.032513	1.30052E-09
*Pinus lambertiana*	72	0.005949	8.26250E-11	0.005264	7.31111E-11
*Pinus taeda*	72	0.007723	1.07264E-10	0.004264	5.92222E-11
*Abies sibirica*	154	0.069804	4.53273E-10	0.054221	3.52084E-10
*Larix sibirica*	133	0.031210	2.34662E-10	0.007401	5.56466E-11
**Pinaceae**	**265**	**0.009825**	**3.70755E-11**	**0.004160**	**1.56981E-11**
***Ginkgo biloba***	**287**	**0.035716**	**1.24446E-10**	**0.007753**	**2.70139E-11**
*Ceratozamia hildae*	41	0.003389	8.26585E-11	0.000312	7.60976E-12
*Zamia integrifolia*	41	0.011686	2.85024E-10	0.009431	2.30024E-10
*Cycas taitungensis*	194	0.003073	1.58402E-11	0.001835	9.45876E-12
**Cycads**	**305**	**0.026821**	**8.79377E-11**	**0.005682**	**1.86295E-11**
*Amborella trichopoda*	117	0.025430	2.17350E-10	0.010369	8.86239E-11
*Liriodendron tulipifera*	117	0.026193	2.23872E-10	0.009041	7.72735E-11

Bold values are summary of each major clade including the taxa sampled within the clade.

Moreover, we calculated the absolute substitution rates R*S* and R*N* per branch by adjusting the d*S* and d*N* by dividing the divergence time of the branch. Again, R*S* and R*N* varied up to 118- and 171-fold among species, respectively, with the highest R*S* and R*N* in Cupressophytes and Gnetophytes, and the lowest in Pinaceae. Within Cupressophytes, R*S* and R*N* varied by 40-fold and 70-fold, with *P. orientalis* having the lowest rates (Table 3). This wide range of substitution rates indicates that sequence evolution is heterogeneous within and among plant lineages, especially in Cupressophytes.

### 3.3 Mitochondrial Gene Loss and Transfer to Nucleus in Cupressophytes and Gnetophytes

Early studies suggest that the mitogenome of the common ancestor of seed plants probably contained 41 protein-coding genes (Richardson et al., 2013; Guo et al., 2016). We found all 41 genes in Cycads, Ginkgo and Pinaceae, but 8–12 genes were absent in Cupressophytes and Gnetophytes (white and orange boxes in Figure 2B). All species in Cupressophytes have lost the same set of eight ribosomal protein genes, while Cupressaceae species and *T. cuspidata* lost an additional *sdh3* gene (Figure 2B). In Gnetophytes, both *G. gnemon* and *W. mirabilis* have lost 11 genes, including 10 ribosomal protein genes and the *sdh3* gene; their *rpl*16 appeared as degraded pseudogene (Figure 2B).

The reduction in mitochondrial protein-coding genes in Cupressophytes and Gnetophytes has been suggested to be a result of gene transfer to the nucleus prior to their loss from the mitogenome (Guo et al., 2016; Guo et al., 2020). To verify whether the lost genes were transferred to the nucleus, we first searched for them in the transcriptomes of Cupressophytes and Gnetophytes (Supplementary Table S2). We recovered 4–7 intact lost mitochondrial genes in transcriptomes of each respective species, but none in *A. heterophylla* (Figure 2B). The recovery of few intact mitochondrial genes in *A. heterophylla* is likely to be a consequence of the limited numbers of ESTs or cDNA sequences in the database, which would hamper detection of these genes in its nuclear genome. We then searched for these recovered genes in their own or related species’ reference genomes and found all of them with high identity (89–100%) and coverage (85–100%) scores (Supplementary Table S3). There were a few exceptions, e.g., *rps*11 and *sdh*3 showed lower identity (52–75%) but high coverage (92–100%) scores, likely due to different substitution rates between genes and species. The BLAST hits of *Podocarpus macrophyllus* genes gave moderate identity scores (48–81%) likely because of the lack of closely related reference. We further analyzed the mapping depth of genome resequencing reads to the mitogenes and transcriptome-recovered genes in each Cupressophytes and Gnetophytes species. In *P. orientalis*, the median depth of read coverage of the 32 genes in mitogenome ranged 275–1,169×, while the nuclear gene *LEAFY* had 15× and the chloroplast *matK* 14,395× (Supplementary Figure S1). The six recovered mitogenes had median depth of read coverage 11–17×, similar to the *LEAFY*, supporting their location in nucleus. Using the same approach, we confirmed that 4–7 of the lost genes in the seven other species of Cupressophytes and Gnetophytes were transferred to the nucleus (orange boxes in Figure 2B). Our results illustrate the differential abundance of nuclear, mitochondrial and plastid genomes in plant cells, and demonstrate the power of depth of read coverage as a quantitative tool for verification of genome origin of a gene.

### 3.4 Sequence Turnover and Structural Rearrangement in Gymnosperm Mitogenomes

**FIGURE 3:**
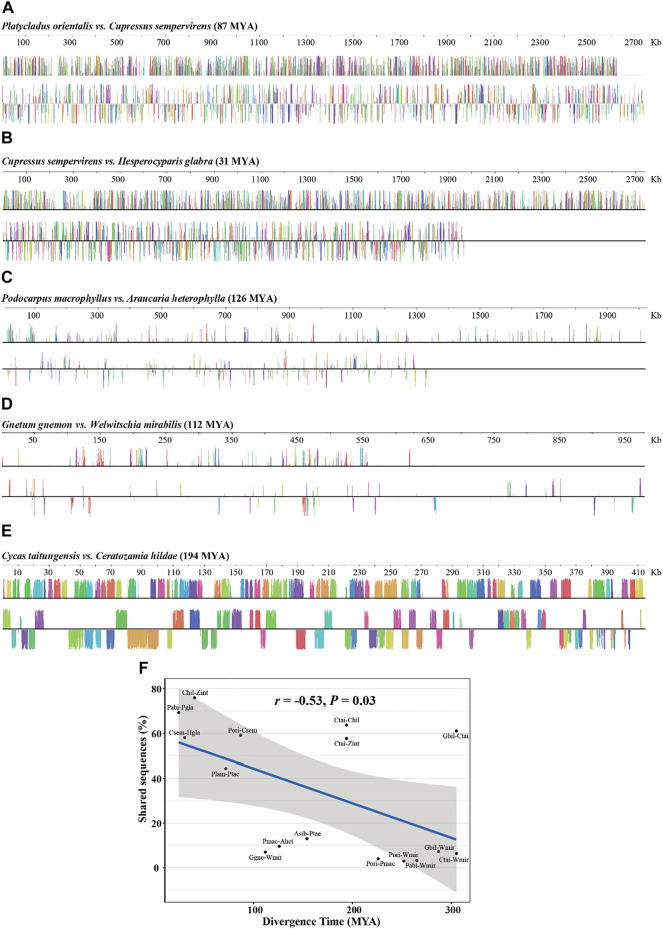
Mitogenome alignments between gymnosperm species showing the locally collinear blocks at varying divergence time. **(A)** *Platycladus orientalis vs. Cupressus sempervirens*. **(B)** *Cupressus sempervirens vs. Hesperocyparis glabra*. **(C)** *Podocarpus macrophyllus vs. Araucaria heterophylla*. **(D)** *Gnetum gnemon vs. Welwitschia mirabilis*. **(E)** *Cycas taitungensis vs. Ceratozamia hildae*. The color bars represent corresponding local collinear blocks, heights are proportional to pairwise sequence identity, and blocks below the center line indicate regions that align in the reverse complement (inverse) orientation. MYA, million years ago. **(F)** Correlation between divergence time and the degree of shared sequences. The blue line represents the regression fitting with a linear model. The grey shadow is the 95% confidence interval of the linear regression.

Rapid structural evolution is thought to be characteristic of plant mitogenomes (Palmer and Herbon, 1988; Cole et al., 2018). To understand the rate of sequence turnover and structural rearrangement in gymnosperms, we examined the degree of synteny and shared DNA among the mitogenomes of the main gymnosperms lineages. We found that syntenic blocks and shared DNA between genomes reduced rapidly with phylogenetic distance (Figures 3A–D; Table 4). For example, *P. orientalis* and *C. sempervirens* diversified for 87 million years (MY) shared ∼60% DNA sequence, but this value dropped sharply to only ∼10% between *P. macrophyllus* and *A. heterophylla* with a divergence time of 126 MY (Figures 3A,C; Table 4). We examined the patterns of shared DNA between 16 pairs of gymnosperm species, and found a strong negative correlation (*r* = 0.53, *p* = 0.03) with time of divergence (Figure 3F). However, as an outlier, exceptionally low DNA turnover was found between *G. biloba* and *C. taitungensis* (Table 4), similar to the report by Guo et al. (2016). In addition, high proportions of shared DNA (58–64%) were retained between *C. taitungensis* and *Ceratozamia hildae*/*Zamia integrifolia* with the divergence time of 194 MY (Figure 3E; Table 4). These results illustrate a variable rate of mitogenome turnover in gymnosperms.

**TABLE 4:** Amount of shared mitochondrial DNA in gymnosperms spanning a wide range of divergence times. MYA, million years ago.

Species compared		Divergence time (MYA)	Shared sequence						
			Length (Kb)			%Genome			
Species 1	Species 2		S1 to S2	S2 to S1	Mean	S1	S2	Mean
*Platycladus orientalis*	*Cupressus sempervirens*	87	1,573	1,599	1,586	60.0	58.3	59.2
*Platycladus orientalis*	*Podocarpus macrophyllus*	226	78	99	88	3.0	4.9	4.0
*Cupressus sempervirens*	*Hesperocyparis glabra*	31	1,311	1,344	1,328	47.8	68.4	58.1
*Podocarpus macrophyllus*	*Araucaria heterophylla*	126	156	157	156	7.7	11.4	9.6
*Gnetum gnemon*	*Welwitschia mirabilis*	112	54	53	54	8.5	5.4	7.0
*Platycladus orientalis*	*Welwitschia mirabilis*	252	31	46	38	1.2	4.7	3.0
*Picea abies*	*Welwitschia mirabilis*	265	39	54	46	0.8	5.6	3.2
*Picea abies*	*Picea glauca*	25	3,808	3,658	3,733	77.7	61.0	69.4
*Pinus lambertiana*	*Pinus taeda*	72	677	855	766	16.9	71.8	44.4
*Abies sibirica*	*Pinus taeda*	154	176	167	172	11.9	14.1	13.0
*Ginkgo biloba*	*Welwitschia mirabilis*	287	37	36	36	10.7	3.7	7.2
*Ginkgo biloba*	*Cycas taitungensis*	305	233	226	230	67.5	54.7	61.1
*Ceratozamia hildae*	*Zamia integrifolia*	41	344	319	332	83.7	68.2	76.0
*Cycas taitungensis*	*Ceratozamia hildae*	194	247	278	262	59.7	67.7	63.7
*Cycas taitungensis*	*Zamia integrifolia*	194	244	265	254	58.8	56.6	57.7
*Cycas taitungensis*	*Welwitschia mirabilis*	305	36	36	36	8.9	3.8	6.4

### 3.5 Repetitive Sequences in Gymnosperm Mitogenomes

**FIGURE 4:**
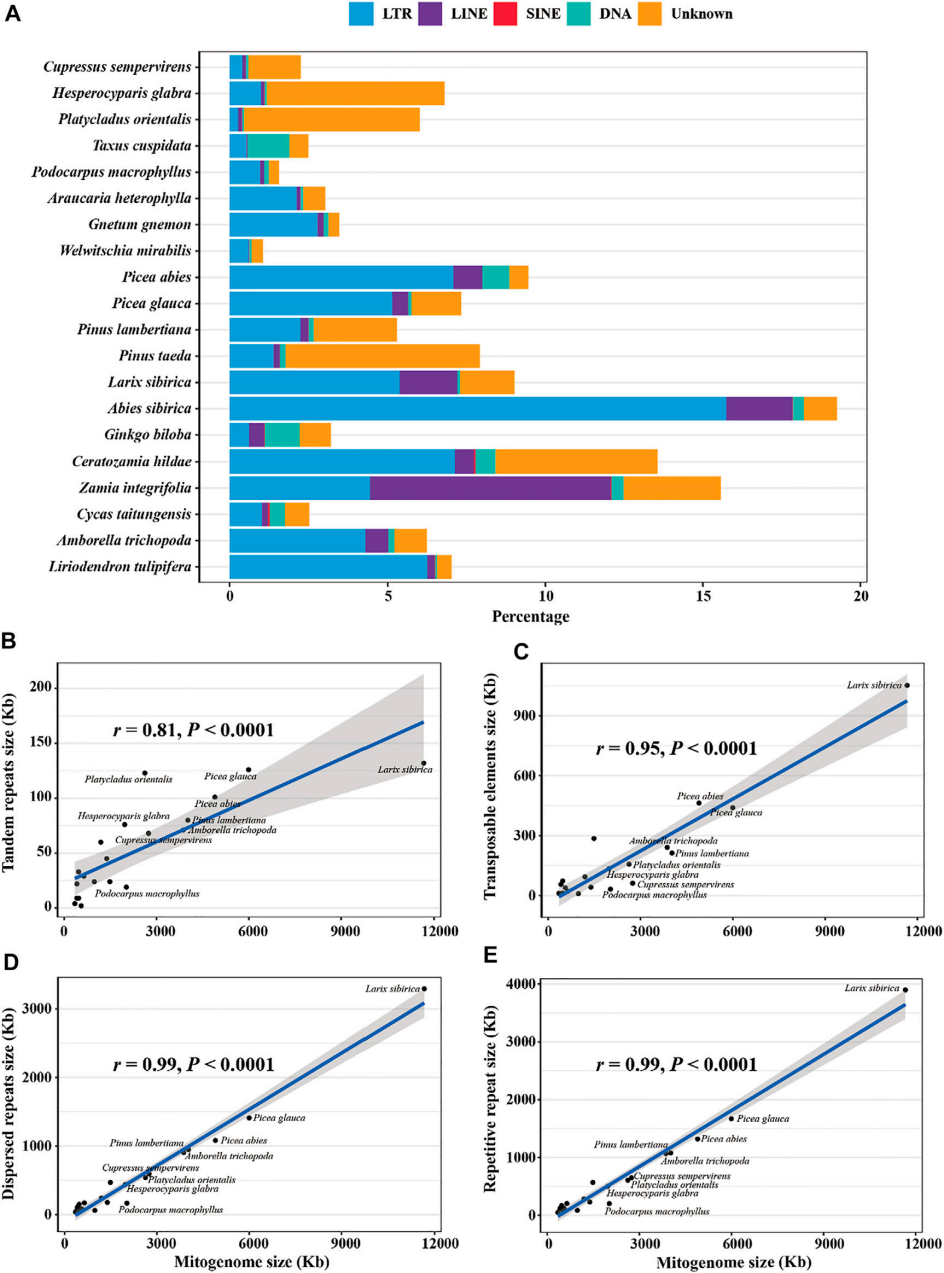
Composition of repetitive sequences in gymnosperm mitogenomes. **(A)** The proportion of TE classes identified in each genome. LTR, long terminal repeat; LINE, long interspersed nuclear element; SINE, short interspersed element; DNA, DNA transposable element; Unknown, unclassified transposable element. **(B)** Correlation between mitogenome size and tandem repeat size, **(C)** TE size, **(D)** dispersed repeat size, and **(E)** repetitive sequence size. The blue line represents the regression fitting with a linear model. The grey shadow is the 95% confidence interval of the linear regression.

Repetitive sequences, particularly TEs, are important components of plant nuclear genomes and contribute to genome size variation and functional regulation (Mehrotra and Goyal, 2014). In contrast, TEs and other repetitive elements in plant mitogenomes are poorly characterized. In this study, we annotated TEs, tandem repeats, and dispersed repeats in the 20 mitogenomes, and found that they have a strong correlation with the genome size and are highly variable among species (Figure 4; Table 5). The highest proportion of TE is in *A. sibirica* (19.26%), followed by *Z. integrifolia* (15.57%), and the lowest in *W. mirabilis* (1.06%). TE contents vary widely even in the same clade, e.g. from 1.58% (*P. macrophyllus*) to 6.80% (*H. glabra*) in Cupressophytes, from 5.29% (*P. lambertiana*) to 19.26% (*A. sibirica*) in Pinaceae, and from 2.52% (*C. taitungensis*) to 15.57% (*Z. integrifolia*) in Cycads. Among TEs, LTRs (long terminal repeats) are the most common group followed by LINE (long interspersed nuclear elements) in most species (Figure 4A). For the three Cupressaceae species and the two *Pinus* species, the most abundant element is the “Unknown” category (Figure 4A). The “Unknown” category of TEs could be due to accumulation of various mutations over long evolutionary time that have degraded the TEs into sequences that cannot be properly classified.

**TABLE 5:** Repetitive sequences in each mitogenome. Dispersed repeats are divided into small-size (<100 bp), medium-size (100–1,000 bp), and large-size (≥1,000 bp) classes. Note, overlapping repetitive sequences are counted only once when calculating their total size.

Species	Repetitive sequences (Kb)				Number of dispersed repeats			
	All	Tandem repeats	Transposable elements	Dispersed repeats	All	Small-size	Medium-size	Large-size
*Platycladus orientalis*	608 (23.18%)	123 (4.70%)	157 (6.00%)	541 (20.60%)	7,634	6,332	1,269	33
*Cupressus sempervirens*	649 (23.66%)	68 (2.48%)	62 (2.26%)	594 (21.65%)	21,162	20,061	1,101	0
*Hesperocyparis glabra*	500 (25.42%)	76 (3.85%)	134 (6.80%)	437 (22.24%)	28,579	26,598	1,823	158
*Taxus cuspidata*	96 (20.45%)	33 (6.99%)	12 (2.49%)	72 (15.30%)	1,157	908	247	2
*Podocarpus macrophyllus*	202 (9.99%)	19 (0.94%)	32 (1.58%)	167 (8.26%)	1,927	1,520	397	10
*Araucaria heterophylla*	230 (16.62%)	45 (3.24%)	42 (3.04%)	178 (12.84%)	2,546	1,817	723	6
*Gnetum gnemon*	203 (31.75%)	29 (4.60%)	22 (3.49%)	171 (26.75%)	22,333	19,944	2,355	34
*Welwitschia mirabilis*	86 (8.83%)	24 (2.49%)	10 (1.06%)	61 (6.22%)	822	781	41	0
*Picea abies*	1,320 (26.95%)	101 (2.07%)	463 (9.46%)	1,082 (22.09%)	14,718	11,856	2,825	37
*Picea glauca*	1,670 (27.86%)	126 (2.10%)	440 (7.33%)	1,411 (23.54%)	19,734	15,853	3,795	86
*Pinus lambertiana*	1,079 (26.82%)	80 (1.99%)	213 (5.29%)	950 (23.63%)	134,961	126,792	8,157	12
*Pinus taeda*	281 (23.58%)	60 (5.04%)	94 (7.93%)	242 (20.30%)	51,637	41,842	9,787	8
*Larix sibirica*	3,899 (33.44%)	132 (1.13%)	1,052 (9.02%)	3,293 (28.23%)	150,913	100,388	50,404	121
*Abies sibirica*	568 (38.24%)	24 (1.60%)	286 (19.26%)	469 (31.54%)	2,712	1,299	1,195	218
*Ginkgo biloba*	49 (14.13%)	4 (1.04%)	11 (3.21%)	38 (10.92%)	2,712	2,685	24	3
*Ceratozamia hildae*	106 (25.84%)	9 (2.24%)	56 (13.56%)	89 (21.72%)	3,054	2,404	643	7
*Zamia integrifolia*	168 (35.76%)	9 (1.95%)	73 (15.57%)	150 (31.92%)	3,390	2,478	831	81
*Cycas taitungensis*	122 (29.30%)	22 (5.40%)	10 (2.52%)	111 (26.75%)	42,039	31,747	10,285	7
*Amborella trichopoda*	1,068 (27.62%)	71 (1.83%)	241 (6.24%)	907 (23.47%)	142,369	113,064	29,249	56
*Liriodendron tulipifera*	116 (20.94%)	2 (0.36%)	39 (7.02%)	83 (15.02%)	669	564	99	6

In addition to TEs, dispersed repeats, including inverted and direct orientation duplicated sequences, are prevalent and made up to 20–30% of the mitogenomes, with a few exceptions (Table 5). The highest proportion is found in Cycads (22–32%) and *A. sibirica* in Pinaceae (32%). The two species in Gnetophytes are highly variable in this regard with 6% in *W. mirabilis* but 27% in *G. gnemon*; this value was stable (21–22%) among the three taxa in Cupressaceae. Tandem repeats are a smaller component compared to dispersed repeats and TEs, and were observed at the level of 1–5% in most of the mitogenomes (Table 5). The overall repetitive sequences composed 20–38% of the analyzed mitogenomes with a few exceptions of 9–17%, and show a strong correlation with genome size (*r* = 0.99, *p* < 0.0001; Figure 4E; Table 5).

### 3.6 Accumulation and Elimination of Dispersed Repeats in Mitogenomes

**FIGURE 5:**
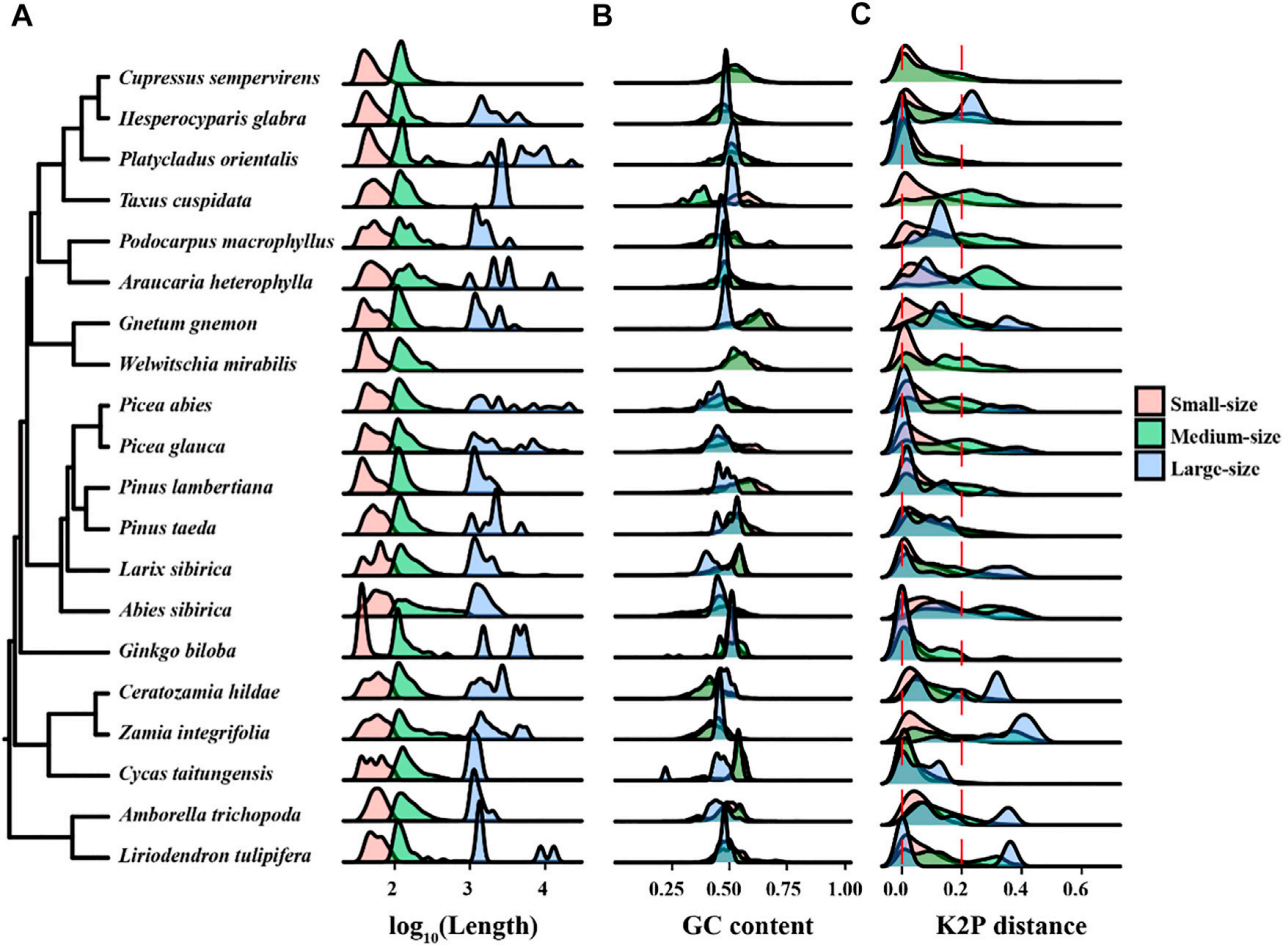
The distribution of repeat size, GC content, and Kimuta 2-parameter (K2P) distance of dispersed repeats in each genome. **(A)** The density plot showing the length distribution of three classes of dispersed repeats. **(B)** The distribution of GC content in each size class. **(C)** The distribution of K2P distances among dispersed repeats. Repeats are grouped into small-size (<100 bp), medium-size (100–1,000 bp) and large-size (≥1,000 bp) classes. The K2P distance of 0 and 0.2 are shown with vertical red dash line**s.** Note, *Taxus cuspidata* contains two large-size dispersed repeats with K2P values of 0 and 0.0046, respectively, which failed to show in the density plot **(C)**

To understand the dynamics of dispersed repeats in mitogenomes, we divided the repeats into small-, medium-, and large-size classes with lengths of <100 bp, 100–1,000 bp, and ≥1,000 bp, respectively. We found that small-size repeats are the most abundant class in the 20 mitogenomes, followed by the medium-size repeats, while large-size repeats are rare (Table 5). The median lengths for the three classes are 52 bp, 136 bp, and 1,517 bp, respectively. The number of small-size repeats are 2–20 fold more common than medium-size repeats; *G. biloba* was extreme having 112-fold more small-size than medium-size repeats (Table 5). In *A. sibirica*, the two classes are balanced. Large-size dispersed repeats are limited, with 50% of the species containing fewer than 15 large repeats (Table 5). On the other hand, three species *A. sibirica, H. glabra* and *L. sibirica*, had more than 100 large repeats (Table 5). Thus, the density plots for small-size and medium-size repeats are similar among species, while it varied widely for the large-size repeats (Figure 5A). The GC contents are relatively conserved for the three classes of dispersed repeats (Figure 5B).

To understand the tempo of evolution in dispersed repeats, we analyzed the distribution of K2P distances among the repeats in the 20 mitogenomes. Small values of K2P represent recent duplications while large values represent more ancient duplication events. We divided the K2P values into three distance classes: ∼0 as recent duplications, >0 and <0.2 as intermediate events, and >0.2 as ancient insertions. A K2P distribution with a dominant narrow peak indicates rapid accumulation of repeats in a short period, otherwise a flattened peak indicates a slow generation of repeats over a prolonged period. As shown in Figure 5C, the K2P distributions of the small-size class is similar among all species with a dominant recent peak and lack of ancient insertions, suggesting they have been accumulated recently over a short time span. In contrast, the K2P distribution of the medium-size and large-size repeats are visibly different among species, often with two or three peaks spanning recent to ancient classes, suggesting multiple and distant expansion events.

Ancient dispersed repeats are easy to identify based on K2P distances, but they are not prevalent in the 20 mitogenomes. This may suggest that the persistance of ancient large-size repeats is governed by the rates of accumulation and elimination. Large repeats can be eliminated or broken down by repeat-mediated recombination, resulting in an increase in small- or medium-size repeats that appear to be more recent expansions (Maréchal and Brisson, 2010; Sullivan et al., 2020).

## 4 Discussion

Assembly of gymnosperm mitogenomes from genomic sequence data is challenging because of complex mechanisms contributing to structural and sequence variation, and lack of well-developed databases and tools for accurate genome mapping. Up to now, only ∼20 gymnosperm mitogenomes have been released. This study contributes a complete mitogenome sequence for Cupressaceae and expands the coverage in gymnosperms. With this new genome, we performed in-depth comparative analyses among major gymnosperm lineages to understand the tempo and mode of mitogenome evolution.

### 4.1 Heterogeneity in Repetitive Elements in Mitogenomes

Among the available gymnosperm mitogenomes, genome sizes vary markedly by 30-fold (Table 1). This large variation is hypothesized to be due to lineage- or species-specific accumulation of repetitive sequences, horizontal transfers and intergenomic transfers (Goremykin et al., 2012; Rice et al., 2013; Guo et al., 2017). It has been reported that 29–100% of sequences in gymnosperm mitogenomes are similar to the nuclear genome sequences (Sullivan et al., 2020). In *P. orientalis* we found 76% undefined sequences (Table 2), which could in part have been derived from the nucleus by intracellular transfer.

Another source of mitogenome size variation is repetitive sequences. Estimations of repetitive contents can be affected by filter and search criteria, e.g. setting the length to 50 bp or 100 bp could underestimate the full size range of repeats. In this study, we searched for dispersed repeats in all size classes. We found that dispersed repeats occupied 6–32% and TEs 1–19% in the sampled mitogenomes. The wide range of dispersed repeats content quantified in this study mirror the results of 1–49% found in 82 angiosperms (Dong et al., 2018). All categories of repetitive sequences are strongly correlated with genome size, giving an overall relationship of *r* = 0.99. Our results illustrate that repetitive sequences, especially TEs and dispersed repeats, are major components of mitogenome expansion.

Although a major component of mitogenomes, the size distributions of repetitive sequences and the dynamics of their accumulation have rarely been investigated in gymnosperms. We analyzed the frequency and sequence diversity of different size classes of repeats, and found small-size repeats are most common while large-size repeats are rare and vary widely among species. K2P distances among repeats indicate that most of the repeats have accumulated by recent expansions. Large-size repeats usually tend to recombine more frequently than small repeats (Skippington et al., 2015; Guo et al., 2016), which may eliminate large repeats over time. However, in *P. abies*, most recombinogenic repeats are small- to medium-size, which deviates from the general expectation that, in the absence of other factors, recombination should scale positively with repeat length (Sullivan et al., 2020). We are of the opinion that genome- or region-specific recombination rates could lead to unique sequence turnover rates resulting in the heterogeneous distribution of large repeats in mitogenomes.

### 4.2 Extensive Rearrangement and Sequence Turnover in Mitogenomes

The plant mitogenome is characterized by rapid structural rearrangement and sequence turnover, driven by a high recombination activity, making mtDNA highly variable even between closely related species (Guo et al., 2016; Cole et al., 2018; Sullivan et al., 2020). In line with this view, extensive rearrangement is found between two spruce species, *P. abies* and *P. glauca* (Sullivan et al., 2020). On the other hand, the mitogenomes of *G. biloba* and *C. taitungensis* have retained substantial amounts of shared DNA despite their divergence time >300 MY (Table 4; Guo et al., 2016). Even with these extreme cases, we found that the synteny blocks and shared DNA between mitogenomes generally decayed with divergence time, with a negative correlation *r* = 0.53 (*p* = 0.03) among the gymnosperms (Figure 3F). Although not exhaustive, our analysis provides a quantitative estimation of the rate of sequence turnover in gymnosperm mitogenomes. Previous studies show that rearrangement in mitogenomes occurs largely through repeat-mediated recombination (Cole et al., 2018; Xia et al., 2020). However, the relationship between inter- and intra-molecule recombination and rearrangement rates among species is unclear (Cole et al., 2018). Recombination dynamics in plant mitogenomes are poorly understood to date and deserve further scrutiny as it drives sequence and structural evolution of plant mitogenomes, and affects genome integrity and biological function.

### 4.3 mtDNA Evidence Support Cupressophytes and Gnetophytes as Sister Groups

The phylogenetic placement of Gnetophytes differs among studies. Five conflicting hypotheses were supported by different marker systems and include: the Gnetophytes-other seed plant hypothesis, the Gnetophytes-other gymnosperm hypothesis, the Gnetifer (Gnetophytes + Conifers) hypothesis, the Gnepine (Gnetophytes + Pinaceae) hypothesis, and the GneCup (Gnetophytes + Cupressophytes) hypothesis (Ran et al., 2018). Yet another suggestion is that Gnetophytes is sister to or within the conifers (Wan et al., 2018). The Gnepine hypothesis is favored by most studies (Ran et al., 2018), including a recent phylogeny based on mitochondrial protein-coding genes (Kan et al., 2021). On the other hand, plastid DNA strongly supports the GneCup hypothesis (Ruhfel et al., 2014; Gitzendanner et al., 2018; Leebens-Mack et al., 2019). This incongruence between datasets might be due to differences in the substitution rates of mitochondrial, plastid and nuclear genes, as well as the amount of informative sites included in each study.

In this study, we inferred the relationships among major gymnosperm lineages using 28 conserved protein-coding genes. The recovered phylogeny supports the GneCup hypothesis with a strong bootstrap support. We are more in favor of GneCup hypothesis because of the shared gene transfer pattern in these two clades. Over evolutionary time, some segments of mtDNA have diverged so much that groups of genes have been lost. The common ancestor of seed plants is inferred to contain 41 protein-coding genes (Guo et al., 2016; Mower, 2020), and Cycads, Ginkgo, and Pinaceae maintained this same set. The mitogenomes of Cupressophytes and Gnetophytes stand out from other gymnosperms in that both have lost 8–11 ribosomal protein-coding genes and the *sdh3* gene, with the exception of *Ephedra przewalskii* (Gnetophytes) which has lost 19 genes (Guo et al., 2020; Kan et al., 2021). To confirm whether these lost genes have been transferred to nucleus, we first annotated them in transcriptomes, then identified their homologs in nuclear genomes, and further examined their depth of mapped reads using genome resequencing data. All three lines of evidence support the transfer of 4–7 mitogenes to nucleus. Our findings are in good agreement with the recent studies by Kan et al. (2020) and Kan et al. (2021), in which they report finding 6–8 intact or partial homologs of lost genes in the nuclear genomes. In sharp contrast, the three other lineages of gymnosperms, including Pinaceae, Ginkgo, and Cycads, share the same set of genes as the common ancestor more than 300 MYA (Guo et al., 2020). The unrecovered missing mitogenes could either be truly lost or not present in the transcriptomes we analyzed due to tissue- and/or time-specific expression, or incomplete annotation from transcriptomes, which identified the genes as partial homologs instead of intact genes.

Other striking characteristics of Cupressophyte and Gnetophyte mtDNA are their accelerated and hugely variable substitution rates across the 28 conserved protein-coding genes relative to other gymnosperms. The synonymous substitution rate d*S* in Cupressophytes and Gnetophytes averaged 0.0641 and 0.0536, respectively. After scaling up by divergence time, the absolute synonymous substitution rates R*S* were similar in Cupressophytes and Gnetophytes with 2.5 × 10^−10^ and 2.1 × 10^−10^ site/year, respectively, and are ∼2–7 fold higher than Pinaceae, Ginkgo, and Cycads. Previous work similarly inferred exceptionally low substitution rates in Pinaceae (*Pinus* and *Picea*) and higher substitution rates in Gnetophytes species (Guo et al., 2016; Sullivan et al., 2020; Kan et al., 2021). However, the extraordinary variation in R*S* in Cupressophytes and Gnetophytes is revealed only by denser sampling of taxa from each clade. Within Cupressophytes, R*S* varied 46-fold among taxa. Our results add to the recognition that while most plant mitogenomes show very low synonymous substitution rates, this rate of divergence is also exceptionally variable for reasons still unclear (Mower et al., 2007). Another unexpected finding is that within Gnetophytes, *G. gnemon* and *W. mirabilis* showed distinct species-specific RNA editing patters as 87–91% of their editing sites differ from each other. Such a striking diversification in RNA editing is not observed within other clades. Future studies with broader taxa sampling are required to understand the mechanisms driving these patterns, including gene loss and transfer, TEs, recombination and selection.

## 5 Conclusion

This study contributes a complete mitogenome assembly of *P. orientalis* to the still very limited accessions in gymnosperms. Our comparative analyses with 19 other mitogenomes of seed plants characterized the composition and distribution of repetitive elements, the tempo of sequence turnover and structural rearrangement, and the frequency of RNA editing in protein-coding genes. Our study revealed shared patterns of cyto-unclear gene transfer and accelerated substitution rates in Cupressophytes and Gnetophytes, and lend support to their sister group placement within the gymnosperms. Our findings highlight and reinforce the dynamic and enigmatic evolution of mitogenomes in gymnosperms.

## Data Availability

The datasets presented in this study can be found in online repositories. The names of the repository/repositories and accession number(s) can be found below: https://www.ncbi.nlm.nih.gov/genbank/, OL703044-OL703045.
