# Supplementary material for: Repetitive Elements, Sequence Turnover and Cyto-Nuclear Gene Transfer in Gymnosperm Mitogenomes
Source: Front Genet. 2022 May 25;13:867736. doi: 10.3389/fgene.2022.867736 (PMC9174605; doi:10.3389/fgene.2022.867736)
Supplement: Supplementary file 1 [file DataSheet1.docx]

**Supplementary Table 1** RNAseq data of *Platycladus orientalis* used for validation of RNA editing sites. **S**ource codes are NCBI accession ID.

| Source | Oligo-dT enriched | Raw reads (Gb) | Clean reads (Gb) |
| --- | --- | --- | --- |
| ERR2040822 | No | 2.3 | 2.2 |
| SRR12349536 | No | 7.4 | 7.0 |
| SRR14381610 | No | 7.0 | 6.3 |
| SRR5892426 | No | 5.5 | 5.0 |

**Supplementary Table 2** Summary of transcriptome assemblies used for recovering of the lost mitochondrial gene. Source codes are NCBI accession ID.

| Species | Source | Oligo-dT enriched | Raw reads (Gb) | Clean reads (Gb) | No. transcripts |
| --- | --- | --- | --- | --- | --- |
| *Platycladus orientalis* | Hu et al., 2016 | Yes | - | - | 148,867 |
| *Cupressus sempervirens* | SRR14381647 | No | 6.8 | 6.5 | 47,512 |
| *Hesperocyparis glabra* | SRR14381630 | No | 7.0 | 6.6 | 55,619 |
| *Taxus cuspidata* | SRR10913923 | Yes | 8.7 | 8.4 | 60,128 |
| *Podocarpus macrophyllus* | SRR14381609, SRR5892448 | No | 15.3 | 14.7 | 71,200 |
| *Araucaria heterophylla* | EST and cDNA (Wegrzyn et al., 2008) | - | - | - | 212 |
| *Gnetum gnemon* | SRR8982868, SRR8982869 | No | 14.6 | 14.2 | 67,306 |
| *Welwitschia mirabilis* | SRR14381587 | No | 6.5 | 6.3 | 58,742 |

**Reference**

Hu, X.G., Liu, H., Jin, Y., Sun, Y.Q., Li, Y., Zhao, W., et al. (2016). *De novo* transcriptome assembly and characterization for the widespread and stress-tolerant conifer *Platycladus orientalis*. *PLoS One* 11(2)**,** e0148985. doi: 10.1371/journal.pone.0148985.

Wegrzyn, J.L., Lee, J.M., Tearse, B.R., and Neale, D.B. (2008). TreeGenes: A forest tree genome database. *Int. J. Plant Genom.* 2008**,** 412875. doi: 10.1155/2008/412875.

**Supplementary Table 3** Summary of blast search of missing mitogenes in reference nuclear genomes

| **Gene name** | **Top hit in the reference genome** | **tblastn identity %** | **tblastn coverage %** | ***E*-value** |
| --- | --- | --- | --- | --- |
| Welwitschia_mirabilis\|rps10 | Welwitschia_mirabilis\|Chr17 | 100 | 100 | 2.97E-76 |
| Welwitschia_mirabilis\|rps13 | Welwitschia_mirabilis\|Chr12 | 98.718 | 100 | 1.13E-42 |
| Welwitschia_mirabilis\|rps14 | Welwitschia_mirabilis\|Chr04 | 89.412 | 99 | 3.79E-42 |
| Welwitschia_mirabilis\|sdh3 | Welwitschia_mirabilis\|Chr05 | 100 | 100 | 3.34E-72 |
| Gnetum_gnemon\|rps2 | Gnetum_montanum\|scaffold843651 | 99.405 | 100 | 1.09E-108 |
| Gnetum_gnemon\|rps10 | Gnetum_montanum\|scaffold703273 | 95.575 | 98 | 5.92E-57 |
| Gnetum_gnemon\|rps13 | Gnetum_montanum\|scaffold919321 | 98.276 | 100 | 4.53E-30 |
| Gnetum_gnemon\|rps14 | Gnetum_montanum\|scaffold688543 | 97.5 | 100 | 3.23E-44 |
| Taxus_cuspidata\|rps1 | Taxus_chinensis\|CM034152.1 | 99.087 | 100 | 2.41E-139 |
| Taxus_cuspidata\|rps2 | Taxus_chinensis\|CM034151.1 | 95.588 | 100 | 6.85E-114 |
| Taxus_cuspidata\|rps7 | Taxus_chinensis\|CM034147.1 | 98.54 | 100 | 2.54E-85 |
| Taxus_cuspidata\|rps10 | Taxus_chinensis\|CM034151.1 | 98.947 | 85 | 1.36E-58 |
| Taxus_cuspidata\|rps14 | Taxus_chinensis\|CM034155.1 | 99 | 100 | 2.28E-57 |
| Taxus_cuspidata\|sdh3 | Taxus_chinensis\|CM034148.1 | 96.063 | 100 | 1.91E-66 |
| Taxus_cuspidata\|rps1 | Taxus_wallichiana\|HiC_scaffold_2 | 100 | 100 | 3.36E-141 |
| Taxus_cuspidata\|rps2 | Taxus_wallichiana\|HiC_scaffold_11 | 95.588 | 100 | 3.63E-113 |
| Taxus_cuspidata\|rps7 | Taxus_wallichiana\|HiC_scaffold_8 | 97.81 | 100 | 1.64E-85 |
| Taxus_cuspidata\|rps10 | Taxus_wallichiana\|HiC_scaffold_41 | 96.842 | 85 | 4.51E-56 |
| Taxus_cuspidata\|rps14 | Taxus_wallichiana\|HiC_scaffold_12 | 98 | 100 | 6.17E-57 |
| Taxus_cuspidata\|sdh3 | Taxus_wallichiana\|HiC_scaffold_4 | 98.425 | 100 | 1.19E-68 |
| Cupressus_sempervirens\|rps1 | Sequoiadendron_giganteum\|chr2 | 86.301 | 100 | 3.12E-123 |
| Cupressus_sempervirens\|rps2 | Sequoiadendron_giganteum\|chr2 | 95.939 | 94 | 5.10E-115 |
| Cupressus_sempervirens\|rps7 | Sequoiadendron_giganteum\|chr9 | 94.891 | 100 | 1.97E-82 |
| Cupressus_sempervirens\|rps10 | Sequoiadendron_giganteum\|chr2 | 91.579 | 85 | 1.27E-55 |
| Cupressus_sempervirens\|rps11 | Sequoiadendron_giganteum\|chr6 | 51.829 | 92 | 3.00E-36 |
| Cupressus_sempervirens\|rps14 | Sequoiadendron_giganteum\|chr9 | 95 | 100 | 2.62E-57 |
| Cupressus_sempervirens\|sdh3 | Sequoiadendron_giganteum\|chr6 | 94.203 | 100 | 2.34E-80 |
| Cupressus_sempervirens\|rps1 | Sequoia_sempervirens\|Scaffold_230435;HRSCAF=248046 | 85.845 | 100 | 9.62E-124 |
| Cupressus_sempervirens\|rps2 | Sequoia_sempervirens\|Scaffold_70574;HRSCAF=78509 | 95.939 | 94 | 2.63E-115 |
| Cupressus_sempervirens\|rps7 | Sequoia_sempervirens\|Scaffold_6215;HRSCAF=7118 | 94.891 | 100 | 5.52E-82 |
| Cupressus_sempervirens\|rps10 | Sequoia_sempervirens\|Scaffold_259073;HRSCAF=277773 | 92.632 | 85 | 5.34E-56 |
| Cupressus_sempervirens\|rps11 | Sequoia_sempervirens\|Scaffold_152400;HRSCAF=166073 | 51.829 | 93 | 1.16E-35 |
| Cupressus_sempervirens\|rps14 | Sequoia_sempervirens\|Scaffold_81965;HRSCAF=90849 | 95 | 100 | 1.72E-56 |
| Cupressus_sempervirens\|sdh3 | Sequoia_sempervirens\|Scaffold_144279;HRSCAF=157463 | 94.203 | 100 | 6.57E-80 |
| Hesperocyparis_glabra\|rps1 | Sequoiadendron_giganteum\|chr2 | 87.671 | 100 | 8.55E-127 |
| Hesperocyparis_glabra\|rps2 | Sequoiadendron_giganteum\|chr2 | 96.447 | 94 | 1.17E-115 |
| Hesperocyparis_glabra\|rps7 | Sequoiadendron_giganteum\|chr9 | 94.891 | 100 | 1.97E-82 |
| Hesperocyparis_glabra\|rps10 | Sequoiadendron_giganteum\|chr2 | 91.579 | 85 | 1.33E-55 |
| Hesperocyparis_glabra\|rps11 | Sequoiadendron_giganteum\|chr6 | 51.829 | 92 | 3.00E-36 |
| Hesperocyparis_glabra\|rps14 | Sequoiadendron_giganteum\|chr9 | 95 | 100 | 2.62E-57 |
| Hesperocyparis_glabra\|sdh3 | Sequoiadendron_giganteum\|chr6 | 94.203 | 100 | 4.15E-80 |
| Hesperocyparis_glabra\|rps1 | Sequoia_sempervirens\|Scaffold_230435;HRSCAF=248046 | 88.128 | 100 | 3.08E-128 |
| Hesperocyparis_glabra\|rps2 | Sequoia_sempervirens\|Scaffold_70574;HRSCAF=78509 | 96.447 | 94 | 6.54E-116 |
|  |  |  |  |  |
| **Supplementary Table 3 continued** | |  |  |  |
| **Gene name** | **Top hit in the reference genome** | **tblastn identity %** | **tblastn coverage %** | ***E*-value** |
| Hesperocyparis_glabra\|rps7 | Sequoia_sempervirens\|Scaffold_6215;HRSCAF=7118 | 94.891 | 100 | 5.52E-82 |
| Hesperocyparis_glabra\|rps10 | Sequoia_sempervirens\|Scaffold_259073;HRSCAF=277773 | 92.632 | 85 | 7.15E-56 |
| Hesperocyparis_glabra\|rps11 | Sequoia_sempervirens\|Scaffold_152400;HRSCAF=166073 | 51.829 | 93 | 1.16E-35 |
| Hesperocyparis_glabra\|rps14 | Sequoia_sempervirens\|Scaffold_81965;HRSCAF=90849 | 95 | 100 | 1.72E-56 |
| Hesperocyparis_glabra\|sdh3 | Sequoia_sempervirens\|Scaffold_144279;HRSCAF=157463 | 94.203 | 100 | 1.16E-79 |
| Platycladus_orientalis\|rps1 | Sequoiadendron_giganteum\|chr2 | 89.041 | 100 | 1.02E-127 |
| Platycladus_orientalis\|rps2 | Sequoiadendron_giganteum\|chr2 | 95.522 | 79 | 2.81E-107 |
| Platycladus_orientalis\|rps7 | Sequoiadendron_giganteum\|chr9 | 96.35 | 100 | 4.01E-84 |
| Platycladus_orientalis\|rps10 | Sequoiadendron_giganteum\|chr2 | 93.684 | 85 | 2.40E-56 |
| Platycladus_orientalis\|rps14 | Sequoiadendron_giganteum\|chr9 | 98 | 100 | 1.80E-58 |
| Platycladus_orientalis\|sdh3 | Sequoiadendron_giganteum\|chr6 | 75.362 | 100 | 1.07E-92 |
| Platycladus_orientalis\|rps1 | Sequoia_sempervirens\|Scaffold_230435;HRSCAF=248046 | 88.584 | 100 | 5.19E-128 |
| Platycladus_orientalis\|rps2 | Sequoia_sempervirens\|Scaffold_70574;HRSCAF=78509 | 88.789 | 87 | 6.95E-109 |
| Platycladus_orientalis\|rps7 | Sequoia_sempervirens\|Scaffold_6215;HRSCAF=7118 | 96.35 | 100 | 1.13E-83 |
| Platycladus_orientalis\|rps10 | Sequoia_sempervirens\|Scaffold_259073;HRSCAF=277773 | 94.737 | 85 | 1.24E-56 |
| Platycladus_orientalis\|rps14 | Sequoia_sempervirens\|Scaffold_81965;HRSCAF=90849 | 97 | 100 | 4.14E-57 |
| Platycladus_orientalis\|sdh3 | Sequoia_sempervirens\|Scaffold_144279;HRSCAF=157463 | 74.879 | 100 | 8.26E-92 |
| Podocarpus_macrophyllus\|rps1 | Taxus_chinensis\|CM034152.1 | 66.364 | 100 | 4.20E-91 |
| Podocarpus_macrophyllus\|rps2 | Taxus_chinensis\|CM034151.1 | 69.388 | 94 | 1.59E-87 |
| Podocarpus_macrophyllus\|rps7 | Taxus_chinensis\|CM034147.1 | 71.91 | 91 | 3.88E-84 |
| Podocarpus_macrophyllus\|rps10 | Taxus_chinensis\|CM034151.1 | 71.134 | 97 | 6.10E-39 |
| Podocarpus_macrophyllus\|rps11 | Taxus_chinensis\|CM034155.1 | 50.649 | 68 | 3.94E-22 |
| Podocarpus_macrophyllus\|rps14 | Taxus_chinensis\|CM034155.1 | 80.612 | 98 | 2.54E-45 |
| Podocarpus_macrophyllus\|rps1 | Taxus_wallichiana\|HiC_scaffold_2 | 66.818 | 100 | 1.28E-91 |
| Podocarpus_macrophyllus\|rps2 | Taxus_wallichiana\|HiC_scaffold_11 | 68.878 | 94 | 4.82E-87 |
| Podocarpus_macrophyllus\|rps7 | Taxus_wallichiana\|HiC_scaffold_8 | 71.348 | 91 | 4.22E-84 |
| Podocarpus_macrophyllus\|rps10 | Taxus_wallichiana\|HiC_scaffold_41 | 69.072 | 97 | 2.83E-37 |
| Podocarpus_macrophyllus\|rps11 | Taxus_wallichiana\|HiC_scaffold_1432 | 80.645 | 91 | 1.14E-11 |
| Podocarpus_macrophyllus\|rps14 | Taxus_wallichiana\|HiC_scaffold_12 | 80.612 | 98 | 2.50E-45 |
| Podocarpus_macrophyllus\|rps1 | Sequoiadendron_giganteum\|chr2 | 60.455 | 100 | 1.19E-80 |
| Podocarpus_macrophyllus\|rps2 | Sequoiadendron_giganteum\|chr5 | 69.388 | 89 | 3.52E-87 |
| Podocarpus_macrophyllus\|rps7 | Sequoiadendron_giganteum\|chr9 | 70.787 | 91 | 1.86E-83 |
| Podocarpus_macrophyllus\|rps10 | Sequoiadendron_giganteum\|chr2 | 70.103 | 97 | 9.28E-39 |
| Podocarpus_macrophyllus\|rps11 | Sequoiadendron_giganteum\|chr6 | 51.22 | 92 | 1.48E-26 |
| Podocarpus_macrophyllus\|rps14 | Sequoiadendron_giganteum\|chr9 | 79 | 100 | 6.10E-45 |
| Podocarpus_macrophyllus\|rps1 | Sequoia_sempervirens\|Scaffold_230435;HRSCAF=248046 | 59.545 | 100 | 3.03E-80 |
| Podocarpus_macrophyllus\|rps2 | Sequoia_sempervirens\|Scaffold_123116;HRSCAF=134943 | 69.388 | 89 | 1.36E-86 |
| Podocarpus_macrophyllus\|rps7 | Sequoia_sempervirens\|Scaffold_6215;HRSCAF=7118 | 70.787 | 91 | 5.23E-83 |
| Podocarpus_macrophyllus\|rps10 | Sequoia_sempervirens\|Scaffold_68257;HRSCAF=75987 | 69.072 | 97 | 1.14E-37 |
| Podocarpus_macrophyllus\|rps11 | Sequoia_sempervirens\|Scaffold_152400;HRSCAF=166073 | 47.651 | 83 | 3.01E-27 |
| Podocarpus_macrophyllus\|rps14 | Sequoia_sempervirens\|Scaffold_81965;HRSCAF=90849 | 81 | 100 | 4.24E-46 |

**Supplementary Table 4** Whole genome resequencing data used for validation the mitochondrial gene transfer. Source codes are NCBI accession ID.

| Species | Source | Raw reads (Gb) | Clean reads (Gb) |
| --- | --- | --- | --- |
| *Cupressus sempervirens* | SRR10713866, SRR10713867 | 9.0 | 7.9 |
| *Hesperocyparis blabra* | SRR10713864,  SRR10713865 | 8.4 | 7.2 |
| *Taxus cuspidata* | SRR10305026 | 37.0 | 31.4 |
| *Podocarpus macrophyllus* | SRR12710837 | 38.7 | 30.0 |
| *Gnetum gnemon* | ERR268421 | 26.2 | 25.9 |
| *Welwitschia mirabilis* | SRR12710831 | 18.6 | 14.9 |

**Supplementary Table 5** Summary of the introns in the protein-coding genes of *Platycladus orientalis* mitogenome.

| Gene | *cis*-spliced intron | *trans*-spliced intron |
| --- | --- | --- |
| atp1 | 0 | 0 |
| atp4 | 0 | 0 |
| atp6 | 0 | 0 |
| atp8 | 0 | 0 |
| atp9 | 0 | 0 |
| ccmB | 0 | 0 |
| ccmC | 0 | 0 |
| ccmFC | 1 | 0 |
| ccmFN | 0 | 0 |
| cob | 0 | 0 |
| cox1 | 0 | 0 |
| cox2 | 0 | 1 |
| cox3 | 0 | 0 |
| matR | 1 | 0 |
| mttB | 0 | 0 |
| nad1 | 0 | 3 |
| nad2 | 1 | 2 |
| nad3 | 0 | 0 |
| nad4 | 0 | 3 |
| nad4L | 0 | 0 |
| nad5 | 2 | 1 |
| nad6 | 0 | 0 |
| nad7 | 0 | 0 |
| nad9 | 0 | 0 |
| rpl5 | 0 | 0 |
| rpl16 | 0 | 0 |
| rps3 | 0 | 0 |
| rps4 | 0 | 0 |
| rps12 | 0 | 0 |
| rps13 | 0 | 0 |
| rps19 | 0 | 0 |
| sdh4 | 0 | 0 |
| Total | 5 | 10 |

**Supplementary Table 6** Comparison of predicted and observed RNA editing sites in *Platycladus orientalis*.

| Gene | Predicted | Observed | Shared |
| --- | --- | --- | --- |
| atp1 | 34 | 39 | 32 |
| atp4 | 19 | 25 | 12 |
| atp6 | 30 | 34 | 27 |
| atp8 | 12 | 8 | 4 |
| atp9 | 18 | 24 | 18 |
| ccmB | 45 | 49 | 35 |
| ccmC | 32 | 35 | 25 |
| ccmFc | 40 | 32 | 18 |
| ccmFn | 65 | 53 | 36 |
| cob | 36 | 52 | 34 |
| cox1 | 57 | 63 | 55 |
| cox2 | 20 | 28 | 18 |
| cox3 | 24 | 42 | 22 |
| matR | 36 | 3 | 2 |
| mttB | 58 | 15 | 8 |
| nad1 | 43 | 49 | 42 |
| nad2 | 52 | 67 | 50 |
| nad3 | 18 | 19 | 13 |
| nad4 | 80 | 100 | 77 |
| nad4L | 21 | 15 | 12 |
| nad5 | 72 | 96 | 59 |
| nad6 | 36 | 46 | 36 |
| nad7 | 25 | 32 | 24 |
| nad9 | 22 | 17 | 13 |
| rpl16 | 5 | 5 | 4 |
| rpl5 | 22 | 20 | 14 |
| rps12 | 10 | 14 | 10 |
| rps13 | 5 | 5 | 4 |
| rps19 | 10 | 9 | 7 |
| rps3 | 25 | 34 | 9 |
| rps4 | 26 | 26 | 20 |
| sdh4 | 9 | 12 | 5 |
| Total | 1,007 | 1,068 | 745 |


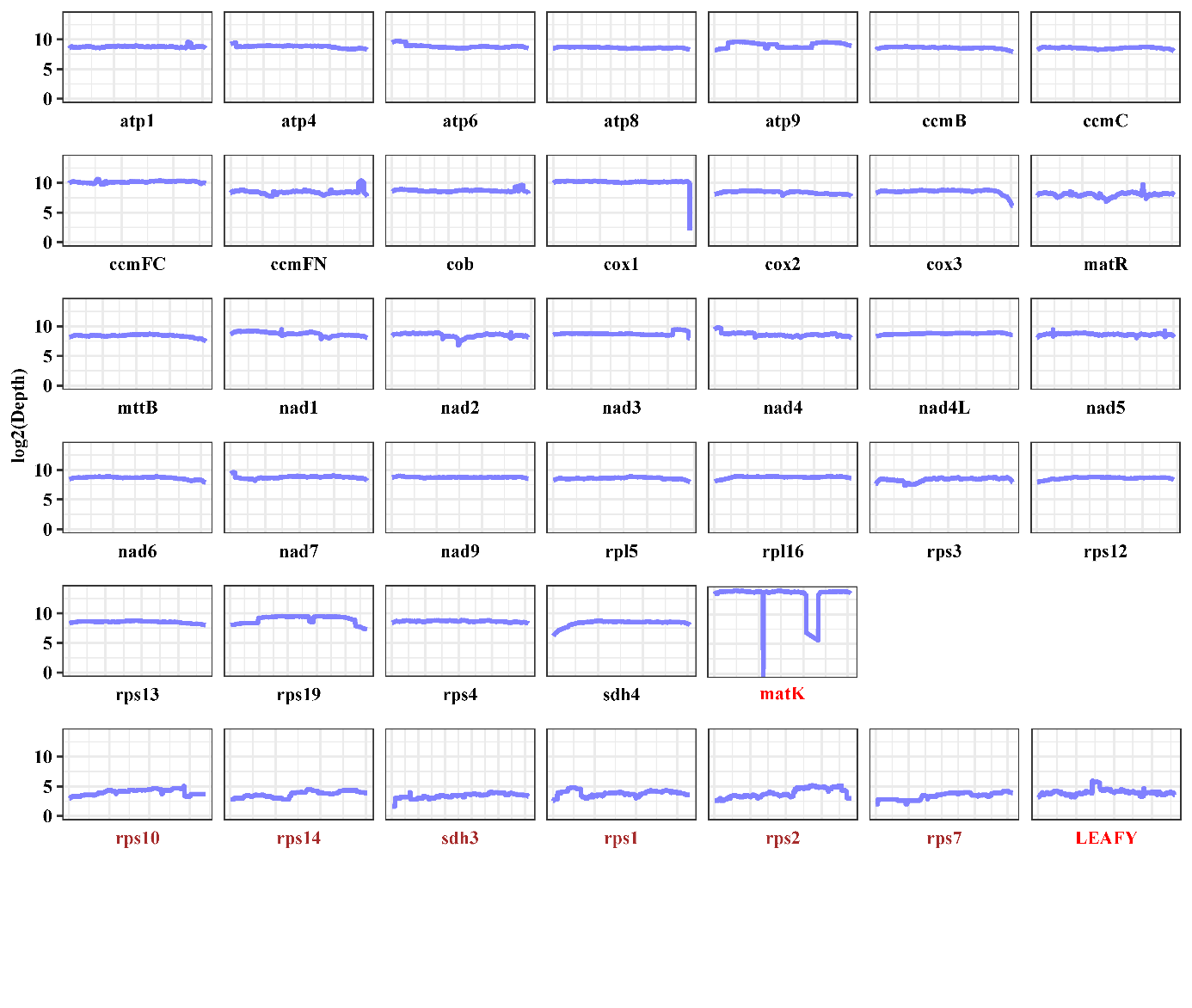


**Supplementary Figure 1** The depth of read coverage of the 32 genes in mitogenome and the 6 transcriptome-recovered mitogenes. The low-copy nuclear gene *LEAFY* and chloroplast gene *matK* were included as spike-in reference. The results lend support for mitogene transfer to nuclear genome.
